# Long‐term survival after adult epilepsy surgery: Mortality and predictors in a large cohort

**DOI:** 10.1111/epi.18564

**Published:** 2025-07-25

**Authors:** Giorgio Fiore, Jane de Tisi, Aidan O'Keeffe, Anna Miserocchi, Andrew W. McEvoy, Josemir W. Sander, John S. Duncan

**Affiliations:** ^1^ UCL Queen Square Institute of Neurology London UK; ^2^ Victor Horsley Department of Neurosurgery National Hospital for Neurology and Neurosurgery London UK; ^3^ Unit of Neurosurgery Fondazione IRCCS Ca’ Granda Ospedale Maggiore Policlinico Milan Italy; ^4^ Department of Pathophysiology and Transplantation University of Milan Milan Italy; ^5^ School of Mathematical Sciences University of Nottingham Nottingham UK; ^6^ UCL Institute of Epidemiology and Health Care London UK; ^7^ Department of Neurology, West China Hospital Sichuan University Chengdu Sichuan China; ^8^ MRI Centre, Chalfont Centre for Epilepsy UK

**Keywords:** epilepsy, mortality, SUDEP, surgery, survival

## Abstract

**Objective:**

Long‐term survival data in adults undergoing surgery for drug‐resistant focal epilepsy remain limited. We examined mortality patterns and predictors in a large cohort followed for over 30 years.

**Methods:**

Adults who underwent epilepsy surgery (1990–2022) were analyzed. Prospectively collected clinical, surgical, and outcome data were included. We estimated the cumulative incidence of mortality and standardized mortality ratios (SMRs) for the cohort. Predictors of epilepsy‐related and non‐epilepsy‐related deaths were identified using Fine–Gray and cause‐specific hazard models.

**Results:**

A total of 1062 individuals contributed 14 279 person‐years of follow‐up (median, 13 years). The overall mortality rate was 6.16 per 1000 person‐years, with 2.52 per 1000 person‐years due to epilepsy‐related deaths, including 0.84 per 1000 person‐years from sudden unexpected death in epilepsy (SUDEP). Epilepsy‐related deaths were more frequent within the first 15 years post‐surgery (*p* = 0.006). The overall SMR was 1.12 (95% confidence interval [CI]: 0.90–1.38), and 0.65 (95% CI: 0.46–0.89) among individuals followed for more than 15 years. Independent predictors of epilepsy‐related mortality were older age at surgery, cortical malformations, and poor post‐operative seizure control (International League Against Epilepsy [ILAE] outcome class ≥4). Non–epilepsy‐related mortality was driven primarily by older age at surgery.

**Significance:**

Epilepsy surgery is associated with reduced premature mortality and increasingly normalized long‐term survival. Age at surgery, post‐operative seizure control, and pathological findings are key determinants of survival, highlighting opportunities to improve surgical outcomes further.

**Key points**

- Epilepsy‐related deaths predominated early after surgery, declining as non–epilepsy‐related causes rise beyond 15 years.
- Over time, long‐term survival aligns increasingly with population‐based expected mortality.
- Older age at surgery, cortical malformations, and poor seizure control independently predicted epilepsy‐related deaths.
- Non–epilepsy‐related mortality was driven primarily by older age at surgery.
- Findings support earlier surgical referral and long‐term risk stratification in drug‐resistant epilepsy.

## 1 INTRODUCTION

Epilepsy imposes a global health burden comparable to lung cancer in men and breast cancer in women.^1^ Premature mortality among people with epilepsy is two‐ to threefold higher than in the general population,^2^, ^3^, ^4^, ^5^ with life expectancy reduced by up to 14 years.^6^ Evidence highlights the protective role of epilepsy surgery over non‐surgical management and the survival advantage of seizure freedom.^1^, ^7^, ^8^, ^9^, ^10^, ^11^ However, most available mortality data for adult surgical cohorts are limited to short‐ or intermediate‐term follow‐up (median follow‐up <10 years). Thus, the long‐term impact of surgery on mortality remains incompletely defined. We present data from a large, long‐term surgical cohort to characterize mortality patterns and identify key predictors, with the aim of optimizing surgical strategies and reducing premature mortality.

## 2 MATERIALS AND METHODS

### 2.1 Study design and participants

Individuals undergoing surgery for drug‐resistant focal epilepsy at the National Hospital for Neurology and Neurosurgery (NHNN), London, UK,^12^ were prospectively followed from February 15, 1990, to November 30, 2022. Comprehensive preoperative assessments determined surgical eligibility,^13^ including detailed clinical histories (epilepsy onset, seizure semiology), physical and neurological examinations, and general medical evaluations for comorbidities. Assessments included electroencephalographic recordings (interictal and scalp video‐EEG [electroencephalography] telemetry) to localize epileptic activity, complemented by high‐resolution magnetic resonance imaging (MRI) for structural abnormalities. Additional imaging techniques, such as fluorodeoxyglucose–positron emission tomography (FDG‐PET), ictal single‐photon emission computed tomography (SPECT), or magnetoencephalography (MEG), were utilized when necessary. Neuropsychological and neuropsychiatric evaluations assessed cognitive and psychiatric profiles, whereas functional imaging (fMRI) or the Wada test were used for language lateralization and motor mapping. Social assessments ensured realistic expectations and adequate support systems. Intracranial EEG was performed in individuals in whom noninvasive methods were inconclusive. Palliative procedures were considered when curative surgery was not feasible. Three epilepsy‐specialized consultant neurosurgeons (William F Harkness [deceased], A.W.McE., and A.M.) conducted the surgical procedures. This work was approved by the Health Research Authority Ethics Committee (22/SC/0016) as a retrospective analysis of anonymized previously acquired data, which does not require individual consent. Reporting followed the Strengthening the Reporting of Observational Studies in Epidemiology (STROBE) guidelines for cohort studies.^14^

### 2.2 Data collection

Data, including demographics (sex, age at onset and at surgery, epilepsy duration) and clinical variables (seizure frequency by seizure type as focal unaware seizure [FUS], focal to bilateral tonic–clonic seizure [FBTCS], and history of status epilepticus) were obtained from a prospectively maintained database (annual outpatient evaluations and direct inquiries by a consultant neurologist [J.S.D.] and a data manager [J.dT.]).^12^ Surgical details (procedure type, laterality, invasive EEG) were recorded, including specifics on the procedure (temporal or extratemporal resection, temporal or extratemporal lesionectomy, functional hemispherectomy, or palliative procedures such as corpus callosotomy or multiple subpial transections). Antiseizure medication (ASM) use was documented preoperatively and at the last follow‐up. Seizure outcome was classified for each postsurgical year (outcome classification [OC]; Table S1) using the International League Against Epilepsy (ILAE) surgery outcome scale.^15^ The distinction between OC 4 and 5 may be challenging and was not analyzed.^12^ Pathological findings included hippocampal sclerosis (HS), vascular malformations, tumors, malformations of cortical development (MCDs), amygdala gliosis, inflammatory changes, epidermoid/dermoid cysts, or negative histology (including infarcts or scars). Mortality data (date and cause) were extracted from the General Register Office (GRO; https://www.gov.uk/general‐register‐office). The senior author reviewed autopsy reports for sudden, unexpected, or cardiovascular deaths. Causes of death concerning epilepsy were categorized as proposed.^15^

### 2.3 Outcomes

The primary aim was to characterize mortality after surgery by examining the cumulative incidence, temporal patterns, and causes of death and by comparing observed mortality with population‐based expectations. We also evaluated predictors of cause‐specific mortality in this population.

### 2.4 Statistical analysis

Descriptive statistics summarized individual characteristics. Continuous variables were reported as means (± standard deviations [SDs]) or medians with interquartile ranges [IQRs], and categorical variables as frequencies and percentages. Kaplan–Meier methods estimated survival. Mortality rates were expressed per 1000 person‐years. Cumulative incidence functions (CIFs) were estimated to examine the temporal distribution of epilepsy‐related deaths (ERDs) and epilepsy‐non‐related deaths (ENRDs) under a competing risks framework. In addition, we examined changes in the proportion of ERDs over time by comparing follow‐up periods of 15 years or shorter and those of longer than 15 years using chi‐square testing. We evaluated whether post‐operative mortality diverges from population expectations by estimating standardized mortality ratios (SMRs) using age‐specific mortality rates from the UK population, as provided by the Human Mortality Database.^16^ SMRs were estimated for the entire cohort and those with more than 15 years of follow‐up. Expected deaths were derived by applying age‐specific rates to observed person‐time. Confidence intervals (CIs) were estimated using the Poisson method.

To identify clinical predictors of mortality, we modeled ERD and ENRD separately. Fine–Gray models estimated subdistribution hazard ratios (SHRs) for cumulative incidence, whereas cause‐specific hazard models (CSHRs) assessed the event rate over time.^17^, ^18^, ^19^ Predictors included age, FBTCS before surgery, histological findings, surgical procedure, ASM number, and OC classes at last follow‐up. Variables that were significant in univariable models were entered into age‐adjusted multivariable models. The proportional hazards assumption was tested using the Schoenfeld test. Those with unknown causes of death were excluded from analyses examining subpopulations of causes.

All tests were two‐sided, with significance set at *p* < 0.05. Analyses were performed using IBM SPSS (version 28.0, International Business Machines Corp, New York, USA) and R software 4.2.2 (R Foundation for Statistical Computing, Vienna, Austria; http://www.r‐project.org/index.html).

## 3 RESULTS

**FIGURE 1:**
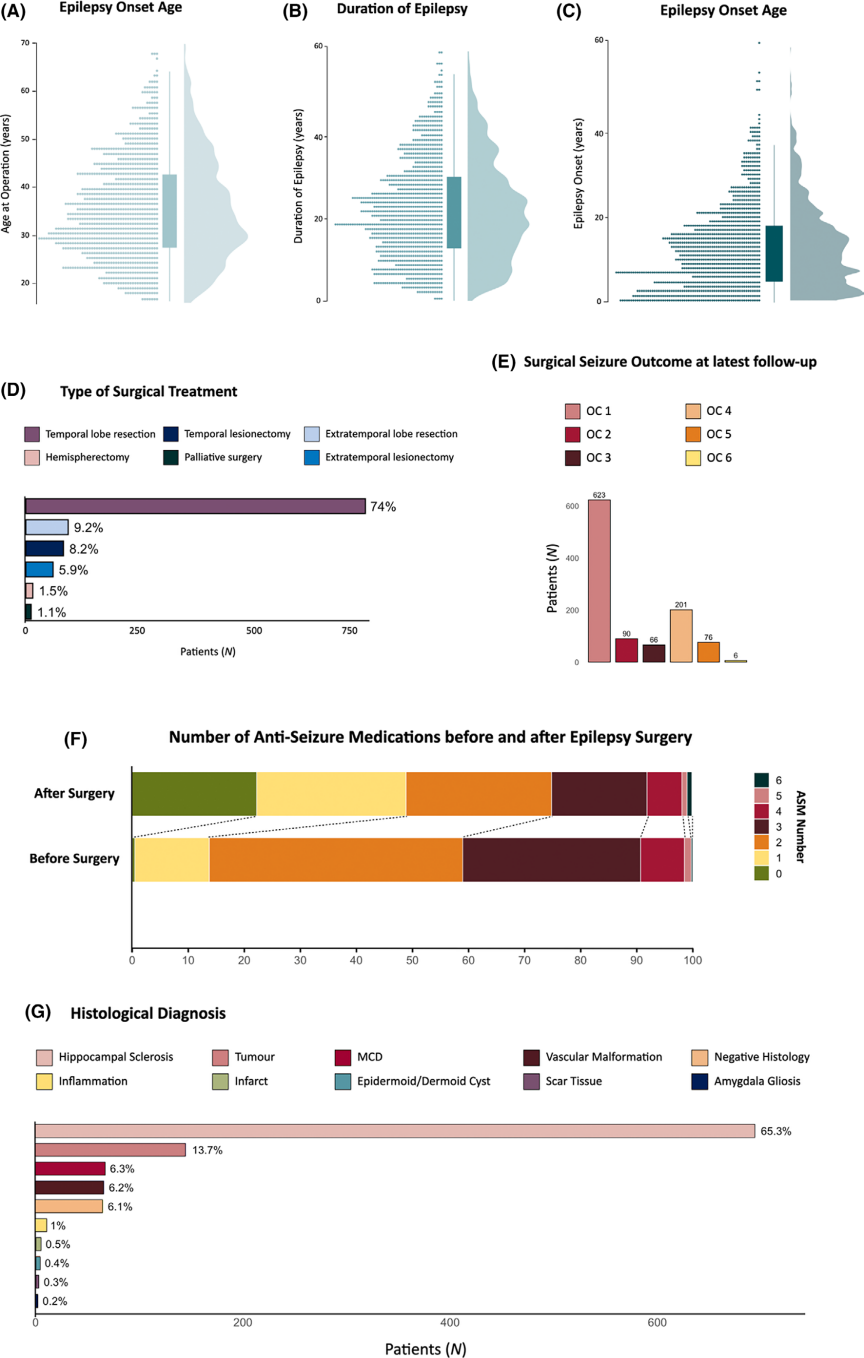
Population characteristics of individuals undergoing epilepsy surgery. (A–C) Distribution of key demographic and clinical variables, including age at operation (A), duration of epilepsy (B), and age at epilepsy onset (C). Histograms depict the overall distribution, with boxplots indicating median and interquartile ranges. (D) Type of surgical treatment performed, with most people undergoing temporal lobe resection (74%). Other surgical approaches include extratemporal lesionectomy, temporal lesionectomy, extratemporal lobe resection, hemispherectomy, and palliative surgery. (E) Surgical seizure outcome at the latest follow‐up, categorized according to the International League Against Epilepsy outcome classification (OC 1–6). (F) Number of antiseizure medications before and after surgery, demonstrating a long‐term reduction in medication burden at the latest follow‐up evaluation. (G) Histological diagnosis of resected tissue, with hippocampal sclerosis being the most common pathology (65.3%). Other diagnoses include tumors, malformations of cortical development, vascular malformations, inflammation, infarcts, epidermoid/dermoid cysts, scar tissue, amygdala gliosis, and cases with no specific histological diagnosis.

One thousand sixty‐two individuals were included, contributing 14 279 person‐years of follow‐up. The median post‐operative follow‐up was 13 years (range 1–33 years), and the mean age ± SD at the last follow‐up was 48.7 ± 11.6 years. The cohort (53% female) had a mean age of 36 ± 10 years at surgery, with a mean epilepsy duration before surgery of 22 ± 12 years. Most individuals underwent temporal surgeries (*n* = 873 [82%]), primarily lobe resections (*n* = 786 [74%]). Invasive preoperative EEG recording was used in 19.6% of cases. In the last annual follow‐up, 58.7% (*n* = 623) were seizure‐free (OC class 1). Only six were classified as OC class 6, so they were pooled with classes 4 and 5 for further analysis. ASM use decreased post‐operatively (χ^2^ = 377, *p* < 2.2e‐16; Figure 1F). Histopathological findings comprised HS in 65.3% of cases, low‐grade epilepsy‐associated tumors (LEATs) in 13.7%, MCDs in 6.3%, and vascular malformations in 6.2%. We analyzed the MCD group (55 with focal cortical dysplasia [FCD] type 2 and 12 with other MCDs) as a whole. Population characteristics are summarized in Figure 1 and Table 1.

**TABLE 1:** Population characteristics.

Variable	*N*	Percentage
*N*	1062	100
Sex (M/F)	499/563	47/53
Post‐operative follow‐up duration, years	14 ± 8	
Onset age, years	12.5 ± 9.8	
Duration of epilepsy, years before surgery	22 ± 12	
Age at operation	36 ± 10	
History of FUS, Y/N	1001/61	94.3/5.7
Frequency of FUS/month in the latest 12 months before surgery	8 (14)	
Invasive EEG recording, Y/N	208/854	19.6/80.4
History of FBTCS at time of operation, Y/N	806/256	75.9/24.1
No history of FBTCS	256	24.1
No history of FBTCS in the latest 12 months before surgery	353	33.2
FBTCS noticed in the latest 12 months before surgery	453	42.7
Frequency of FBTCS /month in the latest 12 months before surgery	4 ± 13	
Preoperative history of status epilepticus, Y/N	136/926	12.8/87.2
Type of surgical treatment		
Side, L/R	561/501	52.8/47.2
Temporal lobe resection	786	74
Temporal lesionectomy	87	8.2
Extratemporal lobe resection	98	9.2
Multilobar resection	8	7.5
Extratemporal lesionectomy	63	5.9
Palliative surgery	12	1.1
Corpus callosotomy	9	0.8
Multiple subpial transections	3	0.3
Hemispherectomy	16	1.5
Number of ASMs at the time of operation		
0	5	0.5
1	141	13.3
2	480	45.2
3	337	31.7
4	83	7.8
5	13	1.2
6	3	0.3
Number of ASMs at the last follow‐up		
0	239	22.5
1	282	26.6
2	276	26
3	180	16.9
4	66	6.2
5	10	0.9
6	9	0.8
Pathology		
HS	694	65.3
HS type 1	506	47.6
HS type 2	38	3.6
HS type 3	69	6.5
Probable HS	23	2.2
Possible HS	58	5.5
Vascular malformation	66	6.2
Cavernomas	59	5.6
AVM	7	0.7
Tumor (LEAT)	145	13.7
DNT	69	6.5
Ganglioglioma	16	1.5
Glioneural	22	2.1
LGG	24	2.3
Other	14	1.3
Malformation of cortical development	67	6.3
FCD 2a/b	53	5
FCD 3	2	0.2
Gray matter heterotopia	2	0.2
Others	10	0.9
Amygdala gliosis	2	0.2
Inflammation	11	1
Epidermoid/dermoid cysts	4	0.4
Other: infarct, scar, or negative histology	73	6.9
OC class at the last follow‐up		
OC 1	623	58.7
OC 2	90	8.5
OC 3	66	6.2
OC 4	201	18.9
OC 5	76	7.2
OC 6	6	0.06

*Note*: Continuous normally distributed variables are reported as mean ± standard deviation (SD). Continuous skewed distributed variables are reported as median (IQR).

Abbreviations: ASM, antiseizure medication; AVM, arteriovenous malformation; DNT, dysembryoplastic neuroepithelial tumor; EEG, electroencephalography; FBTCS, focal to bilateral tonic–clonic seizure; FCD, focal cortical dysplasia; FUS, focal unaware seizure; HS, hippocampal sclerosis; ICEEG, intracranial EEG; IQR, interquartile range; LEAT, long‐term epileptogenic tumor; LGG, low‐grade glioma; OC, outcome classification.

### 3.1 Mortality after surgery

**FIGURE 2:**
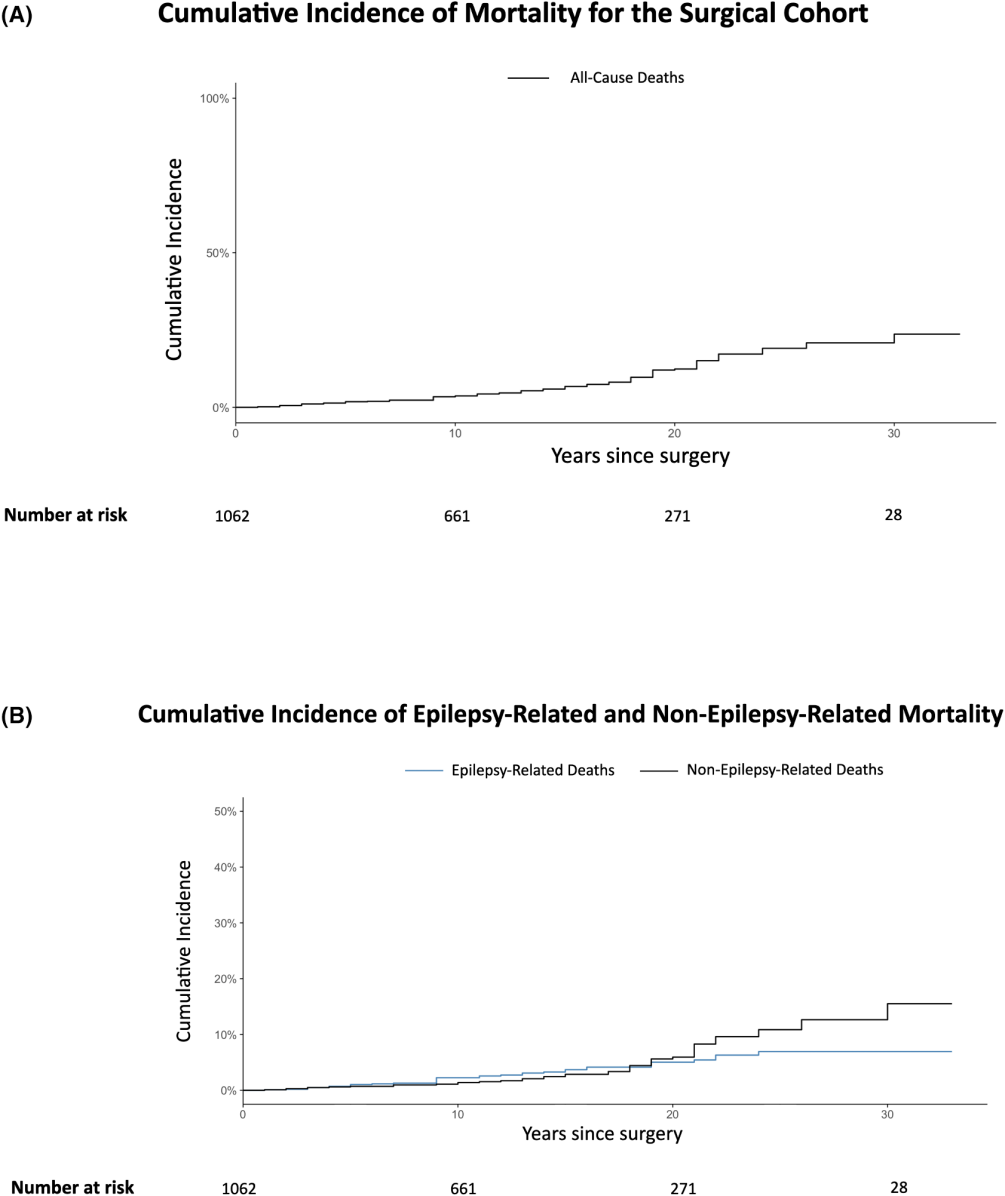
Cumulative incidence of mortality in the surgical cohort. (A) Cumulative incidence of all‐cause mortality following epilepsy surgery. The solid line represents the cumulative probability of death over time, with the x‐axis denoting years since surgery and the y‐axis representing cumulative incidence. The number of people at risk at each time point is displayed below the graph. (B) Competing risk analysis showing the cumulative incidence of epilepsy‐related and epilepsy‐non‐related mortality. The blue line represents epilepsy‐related deaths, whereas the black line represents epilepsy‐non‐related deaths. The x‐axis denotes years since surgery, and the y‐axis represents cumulative incidence probability. The number of individuals at risk at each time point is displayed below the graph.

Eighty‐eight deaths (8.2%) were recorded, representing a mortality rate of 6.16 deaths per 1000 person‐years of follow‐up (Figure 2A). The causes of death are reported in Table S2. Most deaths (45; 3.15 per 1000 person‐years) were unrelated to epilepsy, with cancer (30 deaths; 2.8%) and cardiovascular diseases (9 deaths; 0.8%) being the leading causes. The cause was unknown in seven deaths (0.7%). Those who died of unknown causes were followed for a mean of 14 ± 6 years and had a mean age of 66 ± 11 years at death. Epilepsy‐related deaths accounted for 36 cases (2.52 per 1000 person‐years), including 18 (1.26 per 1000 person‐years) attributed directly to epilepsy (12 sudden unexpected death in epilepsy [SUDEP], 0.84 per 1000 person‐years). The remaining 18 epilepsy‐related deaths (1.7%) included deaths due to underlying neurological disease (*n* = 7, 0.7%), suicide (n = 7, 0.7%), and aspiration pneumonia (*n* = 4, 0.4%). Among the 18 individuals whose deaths were directly attributed to epilepsy, poor medication adherence was documented in two. Living arrangements at the time of demise were known for seven individuals: five lived alone and two with others. Living arrangements could not be reliably determined for the remaining 11.

Individuals who died from epilepsy‐related causes had significantly shorter post‐operative survival than those who died from other causes (median 9 vs 18 years after surgery, *p* = .005; Table S3). Cause‐specific cumulative incidence curves showed a shift in the mortality pattern over time (Figure 2B). Among people followed for more than 15 years (9100 person‐years), epilepsy‐related causes accounted for only 25% of deaths, compared to 75% in those followed for 15 years or less (*p* = 0.006). The long‐term mortality rate in this subgroup was 4.29 per 1000 person‐years. The SMR for the entire cohort was 1.12 (95% CI: 0.90–1.38), indicating no significant excess mortality. Among individuals followed for more than 15 years, the SMR was 0.65 (95% CI: 0.46–0.89), suggesting a significantly lower than expected mortality rate based on general population norms.

### 3.2 Predictors of epilepsy‐related mortality

#### 3.2.1 Subdistribution hazards (Table 2)

**TABLE 2:** Subdistribution hazards of epilepsy‐related mortality.

Variable	Univariable analysis				Multivariable analysis^a^			
	SHR	Lower CI (95%)	Upper CI (95%)	*p* v*a*lue	SHR	Lower CI (95%)	Upper CI (95%)	*p‐* v*a*lue
Age at operation^b^	1.03	1	1.06	0.08	1.04	1.01	1.08	**0.01**
Duration of epilepsy^b^	1.01	0.98	1.04	0.4	–	–	–	–
Age at epilepsy onset^b^	1.02	0.98	1.05	0.3	–	–	–	–
Sex (female)	0.66	0.34	1.26	0.2	–	–	–	–
ICEEG	1.13	0.48	2.66	0.78	–	–	–	–
More than 2 ASMs at last FU	3.74	1.96	7.14	**6.3e** ^**−5**^	1.46	0.6	3.5	0.4
Frequency of FUS	1	1	1	0.13	–	–	–	–
History of FBTCS								
No history of FBTCS	Ref	–	–	–	Ref	–	–	–
Previous history of FBTCS	1.11	0.32	3.9	0.87	0.92	0.27	3.17	0.9
FBTCS in the last 12 months	3.6	1.27	10.3	**0.016**	2.24	0.75	6.7	0.15
Type of surgical treatment								
Temporal lobe resection	Ref	–	–	–	Ref	–	–	–
Temporal Lesionectomy	2.35	0.8	6.87	0.12	1.8	0.5	6.3	0.37
Extratemporal Lesionectomy	0.7	0.09	5.3	0.73	0.4	0.05	3.8	0.44
Extratemporal lobe resection	5.18	2.18	12.26	**0.00019**	1.27	0.36	4.4	0.7
Palliative surgery	15.35	6	39	**9.6e** ^**−9**^	3.6	0.8	15.4	0.09
Hemispherectomy	2.3	0.34	15.9	0.4	2.9	0.42	19.6	0.3
Pathological diagnosis								
HS	ref	–	–	–	ref	–	–	–
Vascular malformation	0.98	0.13	7.5	0.99	0.8	0.1	6.9	0.85
Tumor	2	0.78	5.2	0.15	1.7	0.54	5.2	0.37
Malformation of cortical development	6.97	2.54	19.2	**0.0002**	5.2	1.4	18.9	**0.01**
Amygdala gliosis	–	–	–	–	–	–	–	–
Inflammatory	–	–	–	–	–	–	–	–
Dermoid/epidermoid	–	–	–	–	–	–	–	–
Negative, infarct, or scar tissue	8.08	3.61	18.1	**3.8e** ^**−7**^	2.9	0.4	19.6	0.2
OC class								
OC 1	Ref	–	–	–	Ref	–	–	–
OC 2	1.68	0.36	7.85	0.51	1.6	0.36	7.46	0.5
OC 3	3.57	0.95	13.41	0.06	2.8	0.7	10.8	0.1
OC 4–6	6.05	2.73	13.41	**9.5e** ^**−6**^	2.96	1.04	8.4	**0.04**

*Note*: Statistically significant values are reported in bold.

Abbreviations: ASM, antiseizure medication; CI, confidence interval; EEG, electroencephalography; FBTCS, focal to bilateral tonic–clonic seizure; FU, follow‐up; FUS, focal unaware seizure; HS, hippocampal sclerosis; ICEEG, intracranial EEG; OC, outcome classification; SHR, subdistribution hazard ratio.

^a^ χ^2^ = 56.9, 16 df, *p* = **1.73e** ^**−6**^.

^b^ Increasing age.

Several factors in the Fine–Gray model assessing the incidence of epilepsy‐related mortality were significant in univariable analyses. History of preoperative FBTCS (SHR 3.6, 95% CI 1.3–10.3; *p* = .016), more than two ASMs at last follow‐up (SHR 3.7, 95% CI 1.96–7.1; *p* < .001), extratemporal lobe resections (SHR 5.2, 95% CI 2.18–12.26; *p* < .001), palliative surgery (SHR 15.35, 95% CI 6.00–39.00; *p* < .001), MCD (SHR 6.97, 95% CI 2.54–19.2; *p* < .001; Figure S1A), negative pathology (SHR 8.08, 95% CI 3.6–18.1; *p* < .001; Figure S1A), and poor seizure outcomes at last follow‐up (OC class 4–6; SHR 6.05, 95% CI 2.73–13.4; *p* < .001; Figure S1B) were associated with a higher cumulative incidence of epilepsy‐related mortality.

In the multivariable model, older age at operation (SHR 1.04, 95% CI 1.01–1.08; *p* = .01), MCD (SHR 5.2, 95% CI 1.4–18.9; *p* = .01), and OC class 4 or greater (SHR 2.96, 95% CI 1.04–8.4; *p* = .04) remained independent predictors of cumulative epilepsy‐related mortality. There was no significant association between a preoperative history of FBTCS and surgical type after adjustment for other variables.

#### 3.2.2 Cause‐specific hazards (Table 3)

**TABLE 3:** Cause‐specific hazards of epilepsy‐related mortality.

Variable	Univariable analysis				Schoenfeld test	Multivariable analysis^a^			
	CSHR	Lower CI (95%)	Upper CI (95%)	*p* v*a*lue	*p* v*a*lue	CSHR	Lower CI (95%)	Upper CI (95%)	*p‐* v*a*lue
Age at operation^b^	1.03	1	1.07	**0.05**	0.24	1.05	1.01	1.08	**0.006**
Duration of epilepsy^b^	1.01	0.98	1.04	0.4	0.07	–	–	–	–
Age at epilepsy onset^b^	1.02	0.98	1.05	0.3	0.43	–	–	–	–
Sex (female)	0.65	0.34	1.26	0.2	0.5	–	–	–	–
ICEEG	1.14	0.47	2.75	0.09	0.06	–	–	–	–
More than 2 ASMs at last FU	3.86	2	7.43	**1,00e** ^**−5**^	0.98	1.45	0.68	3.2	0.36
Frequency of FUS	1	1	1.01	0.4	0.38	–	–	–	–
History of FBTCS					0.16				
No history of FBTCS	Ref	–	–	–		Ref	–	–	–
Previous history of FBTCS	1.12	0.32	3.98	0.86		0.9	0.26	3.35	0.9
FBTCS in the last 12 months	3.69	1.29	10.57	**0.015**		2.3	0.78	6.96	0.12
Type of surgical treatment					0.42				
Temporal lobe resection	Ref	–	–	–		Ref	–	–	–
Temporal lesionectomy	2.34	0.8	6.88	0.12		1.7	0.42	7.3	0.45
Extratemporal lesionectomy	0.69	0.09	5.13	0.71		0.4	0.05	3.8	0.44
Extratemporal lobe resection	5.46	2.27	13.12	**0.00015**		1.33	0.4	4.5	0.64
Palliative surgery	16.55	5.6	48.89	**3.84e** ^**−7**^		3.7	0.8	16	0.09
Hemispherectomy	2.36	0.32	17.67	0.4		2.8	0.36	22	0.33
diagnosis					0.96				
HS	Ref	–	–	–		Ref	–	–	–
Vascular malformation	0.97	0.13	7.3	0.97		0.8	0.1	6.7	0.8
Tumor	1.99	0.77	5.12	0.16		1.65	0.4	6.3	0.46
Malformation of cortical development	6.88	2.47	19.1	**0.0002**		5	1.3	19	**0.017**
Amygdala gliosis	0.0001	0	Inf	0.998		0.0001	0	Inf	0.99
Inflammatory	0.0001	0	Inf	0.996		0.0001	0	Inf	0.98
Dermoid/epidermoid	0.0001	0	Inf	0.998		0.0001	0	Inf	0.99
Negative, infarct or scar tissue	8.51	3.71	19.51	**4.23e** ^**−7**^		2.8	0.77	10	0.1
OC class					0.62				
OC 1	Ref	–	–	–		Ref	–	–	–
OC 2	1.68	0.36	7.9	0.51		1.6	0.33	7.65	0.56
OC 3	3.73	0.99	14.1	**0.05**		3.1	0.8	12.1	0.1
OC 4–6	6	2.72	13.6	**1.06e‐5**		2.9	1.12	7.58	**0.029**

*Note*: Statistically significant values are reported in bold.

Abbreviations: ASM, antiseizure medication; CI, confidence interval; CSHR, cause‐specific hazard ratio; EEG, electroencephalography; FBTCS, focal to bilateral tonic–clonic seizure; FU, follow‐up; FUS, focal unaware seizure; HS, hippocampal sclerosis; ICEEG, intracranial EEG; OC, outcome classification.

^a^ χ^2^ = 100.9, 14 df, *p* = **2e** ^**−12**^.

^b^ Increasing age.

The cause‐specific hazard model identified similar predictors of the rate of epilepsy‐related mortality in univariable analysis. Significant factors included older age at operation (CSHR 1.03, 95% CI 1.00–1.07; *p* = .05), preoperative FBTCS (CSHR 3.69, 95% CI 1.29–10.57; *p* = .015), more than two ASMs (CSHR 3.86, 95% CI 2–7.43; *p* < .001), extratemporal resection (CSHR 5.46, 95% CI 2.27–13.1; *p* < .001), palliative surgery (CSHR 16.55, 95% CI 5.60–48.89; *p* < .001), MCD (CSHR 6.88, 95% CI 2.47–19.1; *p* < .001; Figure S2A), negative pathology (CSHR 8.5, 95% CI 3.7–19.5; *p* < .001; Figure S2A), and OC class 4 or greater at the latest follow‐up (CSHR 6, 95% CI 2.7–13.6; *p* < .001; Figure S2B).

Because extratemporal surgeries and MCDs were associated with increased mortality in the univariate analysis, a post hoc analysis was performed to determine whether the excess mortality associated with MCDs was attributable to extratemporal lesions. A CSHR model comparing temporal vs extratemporal MCDs did not suggest a difference (*p* = .76).

In the multivariable cause‐specific model, age at operation (CSHR 1.05, 95% CI 1.01–1.08; *p* = .006), MCD (CSHR 5.00, 95% CI 1.3–19; *p* = .017), and OC class 4 or greater (CSHR 2.90, 95% CI 1.12–7.58; *p* = .029) remained independently associated with an increased rate of epilepsy‐related mortality. The effects of the surgical procedure and negative pathology were no longer significant after adjustment.

## 4 PREDICTORS OF NON‐EPILEPSY‐RELATED MORTALITY

### 4.1 Subdistribution hazards (Table S4)

For ENRD incidence, univariate Fine–Gray models identified significant associations with older age at operation (SHR 1.04, 95% CI 1.02–1.07; *p* = .001), longer epilepsy duration (SHR 1.04, 95% CI 1.01–1.07; *p* = .01), and older age at epilepsy onset (SHR 1.04, 95% CI 1.02–1.07; *p* = .001).

In the multivariate model, only age at operation remained a significant predictor (SHR 2.34, 95% CI 1.7–3.2; *p* < .001). The effects of epilepsy duration and age at onset were no longer significant, suggesting that their influence was mediated by older age at surgery.

#### 4.1.1 Cause‐specific hazards (Table S5)

For epilepsy‐non‐related mortality rate, univariable cause‐specific models revealed significant associations with age at operation (CSHR 1.10, 95% CI 1.07–1.13; *p* < .001), duration of epilepsy (CSHR 1.04, 95% CI 1.01–1.07; *p* = .003), and older epilepsy onset (CSHR 1.05, 95% CI 1.02–1.08; *p* = .001).

In the multivariable model, only age at operation remained an independent predictor (CSHR 1.09, 95% CI 1.06–1.13; *p* < .001). Epilepsy duration and age at onset did not have significant associations with mortality due to collinearity with age.

## 5 DISCUSSION

Mortality following epilepsy surgery remains debated.^1^, ^7^, ^9^ In this long‐term follow‐up of adults undergoing epilepsy surgery, overall mortality was low and comparable to rates reported in population‐based studies^8^ and a meta‐analysis.^9^ However, our SUDEP and ERD rates were lower than those reported in earlier series.^7^, ^8^, ^9^, ^20^ This divergence may reflect differences in cohort composition, individual selection, and structured long‐term follow‐up, underscoring the importance of a systematic approach in improving surgical outcomes. The cohort was highly selected, with a predominance of temporal resections and relatively high rates of seizure freedom. This reflects our long‐standing pre‐surgical philosophy, which places a strong emphasis on aligning surgical decisions with individual expectations and realistic therapeutic goals. Such an approach, although common among specialized centers, may contribute to reduced mortality by optimizing individual selection and post‐operative outcomes. In addition to center‐specific practices, it may also reflect a temporal shift toward non–epilepsy‐related causes of death, more apparent with extended follow‐up. Indeed, ERDs predominated within the first 15 years post‐surgery, whereas afterwards ENRDs became prevalent.

Our SMR analysis further supports the long‐term benefits of epilepsy surgery. Overall mortality was not significantly elevated compared to the age‐matched UK population, and individuals followed for more than 15 years exhibited a mortality rate markedly lower than expected. These findings suggest that successful epilepsy surgery not only reduces epilepsy‐related mortality but may also reshape long‐term survival trajectories to more closely resemble those of the general population.

Mortality risk was not uniform across individuals. Older age at surgery was independently associated with higher epilepsy‐related mortality, even after adjustment for established risk factors—a relationship not previously detailed in surgical cohorts.^7^, ^9^, ^21^ This finding may reflect the cumulative burden of long‐standing epilepsy,^22^, ^23^ which is known to exacerbate structural and functional brain alterations,^24^, ^25^, ^26^ potentially increasing the risk of SUDEP and other epilepsy‐related complications. Alternatively, it may reflect selection bias, with individuals perceived as less likely to benefit from surgery being referred later.^1^ This association reinforces the need for earlier surgical referral and supports its role as a public health strategy to reduce epilepsy‐related mortality.

Seizure outcomes after surgery were strongly associated with mortality. Individuals with suboptimal outcomes had significantly higher mortality, emphasizing the importance of sustained seizure control. These findings build on prior work by moving beyond binary classifications of seizure freedom and highlighting the prognostic value of more granular outcome stratification.^7^, ^8^, ^9^ The reduced rates of ERD observed in the cohort may also reflect favorable seizure control profiles. In multivariable analysis, individuals with poor outcomes (OC 4–6) had nearly a threefold higher risk of epilepsy‐related mortality compared to seizure‐free individuals (OC 1), underscoring that persistent disabling seizures remain a key driver of premature death after surgery, even in well‐selected surgical populations. This reinforces the need for careful individual selection and long‐term outcome monitoring. Although we focused on overall seizure outcomes, the occurrence of post‐operative FBTCS may carry additional prognostic significance.^7^ Future studies should examine whether combining ILAE outcome classification with FBTCS occurrence enhances risk stratification.

The association between MCD, independent of location, and increased mortality supports the role of underlying pathology, likely due to connections to more severe epilepsy networks.^27^, ^28^ Most people with MCD had FCD type 2, which may be associated with more widespread abnormalities, high epileptogenicity, and a broader network that, in some cases, may not be fully addressed by surgery compared to individuals with HS.^12^, ^27^, ^28^

In contrast, variables such as ASM number, pre‐surgical history of FBTCS, and surgical procedure did not independently predict mortality, suggesting that their effects are likely mediated by seizure outcomes or underlying pathology. For instance, individuals requiring more than two ASMs likely represent refractory cases already accounted for by seizure severity. Similarly, higher mortality associated with extratemporal and palliative procedures likely reflects disease complexity rather than the effect of surgery itself.

Age at surgery emerged as the sole independent predictor for non–epilepsy‐related mortality. Epilepsy duration and age at onset were significant in univariate analyses, but lost significance in multivariable modeling. This finding is consistent with previous reports,^8^ suggesting that older age at surgery reduces the risks associated with longer disease duration. The absence of significant associations with other clinical factors suggests that general health factors more strongly influence non‐epilepsy‐related mortality than epilepsy‐specific variables.

Our study has limitations. The cohort was highly selected. The principles of pre‐surgical assessment and surgery at NHNN, established in 1990,^13^ have remained consistent, albeit with adaptation to technological advances. Our practice aligns with that of other major surgery centers, and thus, our results may be generalizable. However, they may not reflect outcomes in centers with broader surgical indications. Replication in other settings would be valuable. The observational nature of this study precluded detailed analysis of the association between older age at surgery and increased risk of epilepsy‐related mortality. Future work should explore whether earlier surgical intervention mitigates this risk. However, ethical considerations and the challenges of achieving comparable follow‐up duration and sample size remain substantial obstacles. Because seizure outcomes were classified using the ILAE outcome scale, our postoperative data distinguished only between focal seizures with and without impaired awareness and did not systematically record FBTCS. This limited our ability to assess the specific impact of FBTCS on mortality, which may be an important prognostic factor. Future studies should address this gap to improve risk modeling. Finally, people with MCD represent a relatively small subgroup within our cohort. The reduced survival rate in this population compared to individuals with HS is notable. Further studies in larger, dedicated MCD populations, with detailed subcategorization, are needed to clarify this association.

Surgery remains an effective strategy to reduce premature mortality in adults with drug‐resistant epilepsy, with benefits that extend beyond seizure control. Our findings highlight that age at surgery, postoperative seizure outcomes, and underlying pathology are key determinants of survival. Notably, long‐term follow‐up demonstrated that overall mortality did not exceed that of the general population and was lower than expected among people followed beyond 15 years. These findings underscore the potential of epilepsy surgery to modify long‐term health trajectories and support the value of early surgical referral and systematic follow‐up in optimizing outcomes.

## 6 CONCLUSION

Surgery is effective in reducing premature mortality for people with epilepsy, shifting the long‐term mortality profile closer to that of the general population. Age at operation, seizure control, and pathological diagnosis emerged as key determinants of survival. These findings highlight the need to strengthen early surgical referral and pathway optimization as part of broader efforts to reduce epilepsy‐related mortality.

## AUTHOR CONTRIBUTIONS

Conceptualization: G.F., J.S.D., J.W.S., and A.M. Data curation: J.dT. and J.S.D. Formal analysis: G.F. and A.O.K. Methodology: G.F., J.S.D., and A.O.K. Project administration: J.dT. Supervision: JSD and JWS. Writing – original draft: G.F., J.S.D. Writing – review & editing: G.F., J.S.D., A.M., A.W.M., and J.S.W. All authors approved the final manuscript, agreeing with the decision to submit it for publication. G.F., J.dT., and J.S.D. directly accessed and verified the underlying data reported in the manuscript.

## FUNDING INFORMATION

This study was supported by the National Institute for Health Research (NIHR) University College London Hospitals/University College London (UCLH/UCL) Biomedical Research Centre, which provided institutional funding for research activities. The sponsor had no role in the study design, data collection, analysis, interpretation, manuscript writing, editing, or the decision to submit the manuscript for publication.

## CONFLICT OF INTEREST STATEMENT

All authors declare that they have no conflicts of interest concerning this work.

## ETHICS STATEMENT

The work was approved by the Health Research Authority Ethics Committee (22/SC/0016) as a retrospective analysis of previously acquired anonymized data, which did not require individual consent. We confirm that we have read the Journal's position on issues involved in ethical publication and affirm that this report is consistent with those guidelines.

## Supporting information


Data S1.

## Data Availability

Anonymized data that support the findings of this study are available from the corresponding author upon reasonable request. J.S.D. had full access to the data and takes responsibility for the integrity and accuracy of the data and the analysis.
