# Supplementary material for: Long‐term survival after adult epilepsy surgery: Mortality and predictors in a large cohort
Source: Epilepsia. 2025 Jul 25;66(11):4198–210. doi: 10.1111/epi.18564 (PMC12661268; doi:10.1111/epi.18564)
Supplement: Supplementary file 1 — Data S1. [file EPI-66-4198-s001.pdf]

Supporting Material

1. **Table S1 Outcome Classification**
2. **Table S2 Cause of Deaths**
3. **Table S3 Median survival comparison by cause of death (deceased patient subgroup)**
4. **Table S4 Subdistribution hazards of epilepsy-non-related mortality**
5. **Table S5 Cause-specific hazards of epilepsy-non-related mortality**
6. **Figure S1 Cumulative Incidence of Epilepsy-Related Mortality by Significant Predictors**
7. **Figure S2 Cause-Specific Hazards of Epilepsy-Related Mortality by Significant Predictors**

Table S1 Outcome Classification

Outcome Class (OC)	Description
OC1	Completely seizure-free
OC2	Only simple partial seizures (SPS); no other seizure types
OC3	Seizures occur on fewer than 4 days per year, with or without SPS
OC4	Seizures on more than 4 days per year, but with ≥50% reduction in seizure days
OC5	No meaningful improvement; seizure days reduced by less than 50% or increased by up to 100%
OC6	Worsening; seizure days increased by more than 100%

As previously reported,[1] seizure outcomes were classified annually using the International League Against Epilepsy (ILAE) surgical outcome scale. The term *SPS* (simple partial seizures) is used throughout to include episodes that may also be described as auras. Outcome class 3 or higher (OC3–OC6) indicates the persistence of seizures beyond SPS alone.

Table S2 Cause of Deaths

**Number of deaths (%)**	88	8.2
**Epilepsy related deaths**	36	3.3
Epilepsy directly related deaths	18	1.69
Sudep	12	1.13
Status Epilepticus	5	0.5
Drowning	1	0.1
Epilepsy indirectly related deaths	18	1.69
Underlying neurological disease	7	0.7
Suicide	7	0.7
Aspiration Pneumonia	4	0.4
**Epilepsy not related deaths**	45	4.2
Cancer	30	2.8
Cardiovascular Disease	9	0.8
Infection	5	0.5
Accident	1	0.1
**Deaths with unknown causes**	7	0.7

Sudep: Sudden Unexpected Death in Epilepsy.

Table S3 Median survival comparison by cause of death (deceased patient subgroup)

	Kaplan-Meier Survival Analysis*		
Group	Median Survival (years)	Lower CI (95%)	Upper CI (95%)
ER Deaths	9	6	11
ENR Deaths	18	15	21

* Log-rank method: p = 0.005; ** Schoenfeld test for proportional hazard assumption= 0.75. CI: confidence interval; ER: Epilepsy related; ENR: Epilepsy not related.

Table S4 Subdistribution hazards of epilepsy-non-related mortality

Variable	Univariable Analysis				Multivariable Analysis*			
	**SHR**	**Lower CI (95%)**	**Upper CI (95%)**	***P value***	**SHR**	**Lower CI (95%)**	**Upper CI (95%)**	***P value***
Age At Operation**	1.04	1.02	1.07	**0.001**	2.34	1.7	3.2	**$2e^{-7}$**
Duration of Epilepsy**	1.04	1.01	1.07	**0.01**	NA	NA	NA	NA***
Age At Epilepsy Onset**	1.04	1.02	1.07	**0.001**	1.12	0.87	1.44	0.36
Sex (female)	0.7	0.39	1.28	0.3	-	-	-	-
ICEEG	1.39	0.65	3	0.4	-	-	-	-
More than 2 ASMs at last FU	1.7	0.9	3.2	0.09				
Frequency of FUAS	0.99	0.97	1	0.09	-	-	-	-
History of FBTCS	1.23	0.84	1.8	0.3	-	-	-	-
Type of surgical treatment	1.19	0.9	1.5	0.15	-	-	-	-
Diagnosis	1.04	0.8	1.4	0.8	-	-	-	-
OC class	1.19	1	1.5	0.1	-	-	-	-

*χ^2 = 35.1, 2 df, p = **$2.39e^{-8}$**; ** Increasing age. Statistically significant values are reported in bold. *** Duration of epilepsy was automatically excluded from the model due to perfect collinearity with age at operation and age at epilepsy onset. These three variables are mathematically related (Duration = Age at operation – Age at onset), and this linear dependency was confirmed through diagnostics in both SPSS and R. Such collinearity results in non-identifiable coefficients in multivariable Cox regression. To ensure valid estimation and preserve interpretability, the final models retained age at operation and age at epilepsy onset, from which duration of epilepsy is derived.

ASM: anti-seizure medication; CI: confidence interval; FBTCS: focal to bilateral tonic-clonic seizures; FUS: focal unaware seizures; ICEEG: intracranial EEG; OC: outcome classification; SHR: Subdistribution Hazard Ratio

Table S5 Cause-specific hazards of epilepsy-non-related mortality

**Variable**	Univariable Analysis				Schoenfeld Test	Multivariable Analysis*			
	**CSHR**	**Lower CI (95%)**	**Upper CI (95%)**	***P value***	***P value***	**CSHR**	**Lower CI (95%)**	**Upper CI (95%)**	***P value***
Age At Operation**	1.1	1.07	1.13	**5e^{-10}*** **	0.017	1.09	1.06	1.13	**3e^{-9}**
Duration of Epilepsy**	1.04	1.01	1.07	**0.003**	0.15	NA	NA	NA	NA****
Age At Epilepsy Onset**	1.05	1.02	1.08	**0.001**	0.5	1.01	0.98	1.03	0.42
Sex (female)	0.7	0.39	1.26	0.2	0.5	-	-	-	-
ICEEG	1.41	0.65	3	0.39	0.29	-	-	-	-
More than 2 ASMs at last FU	1.81	0.98	3.34	0.06***	0.03				
Frequency of FUAS	0.99	0.97	1.01	0.23	0.65	-	-	-	-
History of FBTCS	1.26	0.86	1.8	0.4	0.5	-	-	-	-
Type of surgical treatment	1.14	0.9	1.3	0.09	0.3	-	-	-	-
Diagnosis	1.09	0.9	1.2	0.2	0.87	-	-	-	-
OC class	1.19	0.9	1.4	0.2	0.48	-	-	-	-

*χ^2 = 43.6, 2 df, p = 3.41 e^{-10}; ** Increasing age; *** the conservative Wald method was used to calculate the hazard ratio as the Schoenfeld test was positive; **** Duration of epilepsy was automatically excluded from the model due to perfect collinearity with age at operation and age at epilepsy onset. These three variables are mathematically related (Duration = Age at operation – Age at onset), and this linear dependency was confirmed through diagnostics in both SPSS and R. Such collinearity results in non-identifiable coefficients in multivariable Cox regression. To ensure valid estimation and preserve interpretability, the final models retained age at operation and age at epilepsy onset, from which duration of epilepsy is derived.
ASM: anti-seizure medication; CI: confidence interval; CSHR: cause-specific Hazard Ratio; FBTCS: focal to bilateral tonic-clonic seizures; FUS: focal unaware seizures; ICEEG: intracranial EEG; OC: outcome classification.

Figure S1 Cumulative Incidence of Epilepsy-Related Mortality by Significant Predictors

A Cumulative incidence of epilepsy-related deaths by pathology

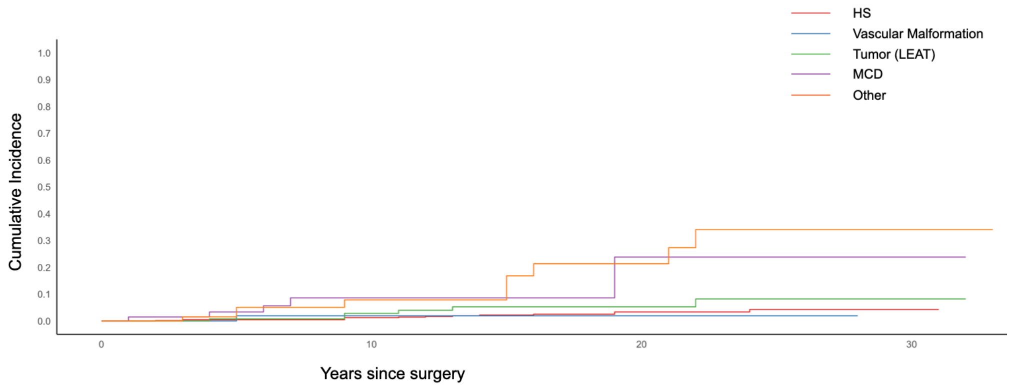

Number at risk

HS	689	470	193	16
Vascular Malformation	65	31	11	0
Tumor (LEAT)	144	90	42	9
MCD	67	21	4	1
Other	90	43	21	2

B Cumulative incidence of epilepsy-related deaths by outcome classification (OC) classes

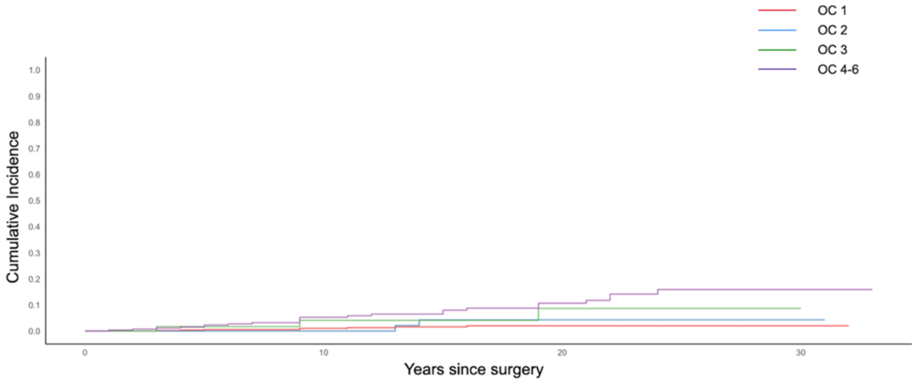

Number at risk

OC 1	621	394	145	12
OC 2	90	53	26	4
OC 3	65	38	16	1
OC 4-6	279	170	84	11

(A) Cumulative incidence of epilepsy-related mortality stratified by histopathological diagnosis. The x-axis represents years since surgery, and the y-axis represents cumulative incidence. Histopathological categories include hippocampal sclerosis (HS), vascular malformations, tumour-associated epilepsy (LEAT), malformations of cortical development (MCD), and negative pathology/infarct/gliosis. The number of patients at risk at each time point is displayed below the graph. (B) Cumulative incidence of epilepsy-related mortality stratified by postsurgical seizure outcome classification (OC). The x-axis represents years since surgery, and the y-axis represents cumulative incidence. Seizure outcome categories include OC 1, OC 2, OC 3, and OC 4–6. The number of patients at risk at each time point is displayed below the graph.

Figure S2 Cause-Specific Hazards of Epilepsy-Related Mortality by Significant Predictors

A Cumulative hazards of epilepsy-related deaths by pathology

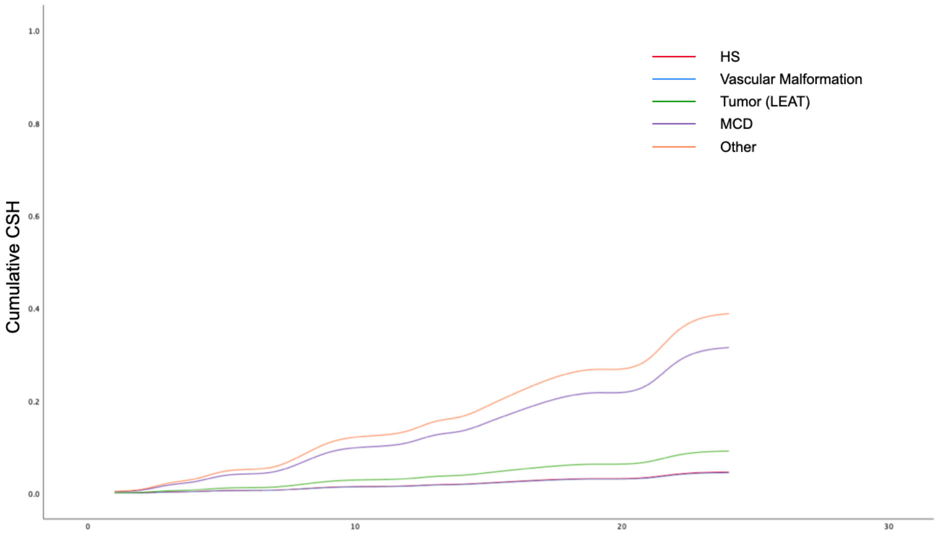

Number at risk			
HS	689	470	193
Vascular Malformation	65	31	11
Tumor (LEAT)	144	90	42
MCD	67	21	4
Other	90	43	21

B Cumulative hazards of epilepsy-related deaths by outcome classification (OC) classes

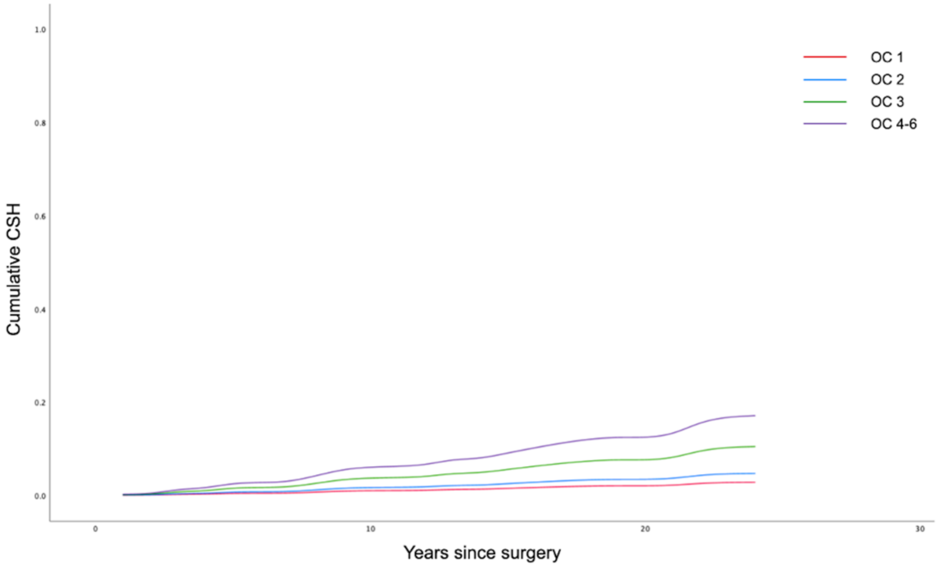

Number at risk			
OC 1	621	394	145
OC 2	90	53	26
OC 3	65	38	16
OC 4-6	279	170	84

(A) Cumulative cause-specific hazard of epilepsy-related mortality stratified by histopathological diagnosis. The x-axis represents years since surgery, and the y-axis represents the cumulative cause-specific hazard. Histopathological categories include hippocampal sclerosis (HS), vascular malformations, tumour-associated epilepsy (LEAT), malformations of cortical development (MCD), and negative pathology/infarct/gliosis. The number of patients at risk at each time point is displayed below the graph. (B) Cumulative cause-specific hazard of epilepsy-related mortality stratified by postsurgical seizure outcome classification (OC). The x-axis represents years since surgery, and the y-axis represents the cumulative cause-specific hazard.

Seizure outcome categories include OC 1 (seizure freedom), OC 2, OC 3, and OC 4–6. The number of patients at risk at each time point is displayed below the graph.

References

1. De Tisi J, Bell GS, Peacock JL, McEvoy AW, Harkness WF, Sander JW, et al. The long-term outcome of adult epilepsy surgery, patterns of seizure remission, and relapse: A cohort study. The Lancet. 2011; 378(9800):1388–95.
